# Epilepsy in patients with Alzheimer's disease: A systematic review

**DOI:** 10.1590/S1980-57642014DN81000010

**Published:** 2014

**Authors:** Diane da Costa Miranda, Sonia Maria Dozzi Brucki

**Affiliations:** 1 MD, Resident of Neurology, Hospital Santa Marcelina, São Paulo, Brazil;; 2 PhD, Neurologist, Hospital Santa Marcelina; Cognitive and Behavioral Neurology Unit, University of São Paulo, São Paulo, Brazil.

**Keywords:** Alzheimer's disease, epilepsy, dementia, antiepileptic drugs

## Abstract

Alzheimer's disease (AD) and epilepsy are common disorders in the elderly. Evidence demonstrates that patients with AD have an increased risk of developing epilepsy and seizures.

**Objective:**

To review epidemiological, clinical and treatment aspects of epilepsy and AD.

**Methods:**

We reviewed databases (PubMED, LiLACS, Scielo) conducting a search for manuscripts using the terms Alzheimer's disease and epilepsy.

**Results:**

Manuscripts related to the areas of interest were reviewed. Studies revealed that epilepsy is more frequent among AD patients. The combined presence of the two disorders may be related to mechanisms of neuronal hyperexcitability as a consequence of amyloid-beta protein (Aβ) or phosphorylated tau accumulation, as well as to structural changes in cortical and hippocampal regions. Available data suggest that the new generation of antiepileptic drugs (AEDs) are better tolerated in the elderly population, and may also be the best option in patients with AD and epilepsy.

**Conclusion:**

Further prospective studies involving evaluation of concomitant dementia and epilepsy, neurophysiological findings and biomarkers need to be performed.

## Abstract (pt)

Doença de Alzheimer (DA) e Epilepsia são desordens frequentemente vistas em idosos. Evidências mostram que pacientes com DA possuem um risco aumentado de desenvolver crises e epilepsia.

**Objetivo:**

Rever aspectos epidemiológicos, clínicos e de tratamento relacionados à DA e epilepsia.

**Métodos:**

Nós revimos as bases de dados (PubMED, LiLACS, Scielo) procurando por manuscritos com termos: Doença de Alzheimer e epilepsia.

**Resultados:**

Manuscritos que continham assuntos de nossa area de interesse foram revisados. Os estudos mostram que a frequência de epilepsia é maior em pacientes com DA, possivelmente ligados à deposição de beta-amiloide e proteína tau, que aumentam a hiperexcitabilidade neuronal, bem como alterações estruturais na região hipocampal. Dados disponíveis sugerem que as drogas antiepilépticas de nova geração são mais bem toleradas na população idosa, e talvez também sejam a melhor escolha nos pacientes com DA e epilepsia.

**Conclusão:**

Mais estudos prospectivos com evolução de demência e epilepsia concomitantes com achados neurofisiológicos e de biomarcadores necessitam ser desenvolvidos.

## INTRODUCTION

Epilepsy is a neurological disorder defined in the 1989 epilepsy classification as the occurrence of at least two unprovoked seizures (seizures which occur without any obvious immediate cause, in this case called acute symptomatic seizures) which are clinical manifestations of abnormal and excessive neuronal discharge that may cause varied, sudden and transient symptoms including altered consciousness and/or motor, sensory or psychiatric events.^1,2^

It is known that the incidence of seizures and epilepsy increases with age and many factors are related to the onset of seizures in the elderly: degenerative diseases (such as Alzheimer's disease [AD]), strokes, tumors (primary or metastatic), head injuries (mainly subdural hematomas), metabolic disorders, infections, and the use of medications that may lower seizure threshold.^2-5^

Evidence derived from population-based and observational studies and, more recently, from studies using animal models, show that AD is associated with a substantially increased risk of seizures and epilepsy in the elderly.^1,3,6,7^ However, the actual relationship between these two disorders requires further research.

Moreover, there are many difficulties in establishing the epilepsy diagnosis in this group of patients, since confirmation of a seizure requires a consistent clinical history which is difficult to achieve in patients with a dementia condition owing to difficulties remembering and/or reporting the event.^1^ In addition, it is a challenge to differentiate an ictal symptom from behavioral symptoms of dementia (fluctuating level of consciousness and attention, hallucinations, confusion).^4^

This systematic review aims to outline relevant aspects of epilepsy in patients with AD, since with a better understanding of the mechanisms behind this combination of conditions, it may be possible to determine the role of epileptiform abnormalities in cognitive impairment and disease progression. Consequently, this may allow the identification of new therapeutic targets and contribute toward improving the current scenario. Manuscripts on epidemiology, clinical aspects, and pathophysiology were focused.

## METHODS

The study was conducted based on the national and international electronic databases, LILACS, SciELO and PubMed using "Alzheimer's Disease" and "Epilepsy" as key words. Complete articles published in the 2004-2014 period written in the English and Portuguese languages were retrieved.

## RESULTS

Using epilepsy and Alzheimer's disease for the selected search period (last ten years) retrieved 996 articles. Only 66 were selected after careful evaluation of the abstract, covering the defined target areas. After removing the redundant references listed in more than one database, the articles were read and evaluated. Only those addressing the areas comprised in this study approach were include, giving a final total of 30 articles. We selected articles according to the areas of interest as described below:

**Epidemiology.** Studies showed that the prevalence and incidence rates of epilepsy are higher in demented than non-demented patients.

In the study by Amatniek et al. (2006), patients with moderate AD were followed prospectively for a period of six months, revealing an epilepsy incidence of 0.87% (12/1,374 person years observed) with higher rates found in the younger group. In the UK, a case-control study showed an incidence of 5.6/1,000 people/year (95% CI 4.6-6.9) in AD and 0.8/1,000 people/year (95% CI 0.6-1.1) in the group without dementia. Two cohorts investigated in the US, showed an incidence of 484/100,000 people/year (95% CI, 287-764) and 418/100,000 people/year, respectively (Table 1).^8-11^

**Table 1:** Incidence and predictors of seizures in patients with AD from selected studies.

Author and publication year	Country	Type of study	Incidence	Risk Factors
Amatniek JC et al., 2006	USA	Prospective(233 Mild AD patients were prospectively followed at 6-month intervals)	0.87% (12 seizures events/1,374 py)	• Younger age (RR, 0.89 per year increase in age; 95% CI, 0.82-0.97)
				• African-American ethnic background (RR, 7.35; 95%CI, 1.42-37.98)
				• More severe dementia (RR, 4.15;95% CI, 1.06-16.27)
				• Focal epileptiform findings on EEG(RR, 73.36; 95% CI, 1.75-3075.25)
Imfeld P, 2013	United Kingdom	Case control(7,086 patients with AD and 11,524 matched controls without dementia diagnosis)	5.6/1,000 py (95% CI 4.6-6.9) in AD and 0.8/1,000 py (95% CI 0.6-1.1) in dementia-free group	• Longer standing (>3 years) history of diagnosed AD (OR, 10.7; 95% CI, 5.4-21.4)
Irizarry MC et al., 2012	USA	Cohort (3078 subjects randomized to the treatment or placebo arms of 10 AD clinical trials)	484 per 100 000 py (95% CI, 287-764)	• Younger age (HR, 0.80; 95% CI, 0.70-0.91 for every 5 years of age)
				• Younger age at dementia onset (HR, 0.79; 95% CI, 0.70-0.90 for every 5 years of age)
				• Lower MMSE score (HR, 3.9; 95% CI, 1.5-10.1 for MMSE scores <18 compared with MMSE scores ≥18)
				• Memantine use (HR, 3.87; 95% CI, 1.12-13.49), and antipsychotic use (HR, 4.0; 95% CI 1.5-10.3)
Scarmeas N et al.,2009	USA	Cohort (453 patients with probable AD observed prospectively from mild disease stages since 1992)	418 per 100 000 py	• Younger age (HR for each additional year of age, 1.23; 95% CI, 1.08-1.41)

py: person-years; CI: confidence interval; HR: hazard ratio; MMSE: Mini-Mental State Examination; RR: relative risk; EEG: electroencephalogram; OR: Odds ratio.

The reported prevalence of epilepsy in AD ranges from 10% to 22%.^1,2,12^ However, methodological problems limit the accuracy of these data because the samples in studies with pathological confirmation of AD are both small and vary widely in duration and severity of disease. Moreover, many prospective and retrospective studies include other forms of dementia besides symptomatic causes of epilepsy.^4^

Some predictors of seizures were found in association with AD: early onset of dementia, advanced stage of the disease,^4,12^ African-American ethnicity, focal epileptiform findings on the electroencephalogram (EEG), low score on the Mini-Mental State Examination (MMSE), use of memantine and antipsychotic drugs^9,10^ (Table 1). Furthermore, evidence suggests that genetic forms of AD may also be associated with an increased risk of epilepsy.^3,13^

**Clinical aspects.** The correct classification of seizure type is an important step in the management of any patient with epilepsy. It is particularly complicated in patients with dementia due to their cognitive impairment that prevents them from remembering and even reporting the event occurred.^1^

There are few studies in the literature evaluating what type of seizures happen more often in AD. It was stated that both generalized seizures and complex partial seizures are likely to occur, but partial seizures with/without secondary generalization tend to predominate.^1-3,13-15^

*The role of EEG* - Patients with AD may exhibit no epileptiform abnormalities such as slowed theta and delta on the EEG. However, there is little evidence on the presence of spicules or sharp waves and their prognostic value in the development of seizures.^4^ In a study of 1674 patients with AD and other dementias, surface EEG detected epileptiform discharges in only 42 (3%) patients, similar rates to those found in the general population.^4^ A prospective cohort study conducted the EEG in only some of the subjects with AD (5%) and 16% of whom had epileptiform activity.^11^ However, in a retrospective study that included broad forms of dementia, EEG was obtained for 29 patients (75%) of whom 15 (38%) had epileptiform discharges, most commonly unilateral or bitemporal.^16^

**Possible mechanisms.** In AD, the underlying mechanisms of the emergence of seizures remain uncertain. Among the proposed mechanisms, the accumulation of amyloid-beta (Aβ), neurofibrillary tangles as well as pathological changes in mesial temporal structures including CA1, subiculum and entorhinal cortex stand out.^2,3,12,17^

*A*β *accumulation* - One of the hallmarks of AD is the deposition of Aβ plaques, which are considered the result of proteolytic cleavage of the amyloid precursor protein (APP). It is still unclear how Aβ deposits contribute to neuronal damage, but it is known that exposure to pathologically relevant levels of Aβ induces hyperexcitability in individual neurons and neural circuits.^12,18-21^ Models in transgenic animals, such as mice expressing human amyloid precursor protein (HAPP) with high levels of Aβ, develop not only AD-like pathological changes and cognitive deficits, but spontaneous epileptiform activity has also been shown in cortex and hippocampus.^18-20,22^

To strengthen the hypothesis, researchers monitored the cortical and hippocampal neuronal activity using video electroencephalography (video-EEG) in HAPP mice that still had no evident neuronal loss. The video showed frequent epileptiform activity including spicules and sharp waves suggesting that high levels of Aβ are sufficient to elicit epileptiform activity *in vivo* in the absence of overt degeneration.^18^

Minkeviciene et al. (2009) used HAPP mice (APdE9) and compared them with their nontransgenic littermates. Twenty APdE9 and ten non-transgenic mice received an implant of electrodes for the video-EEG. During the first week, five out of the 20 APdE9 mice (25%) had at least one electrographic seizure. None of the control group animalis exhibited epileptiform activity. In the third week, the proportion of APdE9 mice with seizures increased to 11 out of 20 (55%). Moreover, the recordings of the brain electrophysiological layers of these mice showed increased excitability and abnormal depolarization of the membrane potential in cortical pyramidal cells.^23^

*Neurofibrillary tangles (NFT)* - The tau protein is a member of the family of proteins associated with microtubules. During abnormal conditions, the tau can become hyperphosphorylated and accumulate in NFT, resulting in a group of disorders known as tauopathies, the most common of which is AD.^24,25,26^ A new role of this tau has emerged, suggesting that tau might be involved in the regulation of neuronal hyperexcitability.^24,25^ This was exemplified in an experimental study testing the effect of tau reduction in non-transgenic adult mice by reducing endogenous tau levels using an antisense oligonucleotide infused into cerebrospinal fluid and subsequently analyzing the effects on behavior and induction of seizures. When tau was reduced in the mice, no change was observed in motor and memory/learning tasks, but regarding chemically-induced seizures, this tau reduction interfered in seizure severity.^24^

*Other hypotheses* - Models of temporal lobe epilepsy suggest that cell loss and reorganization of neuronal circuitry in the mesial temporal regions leads to pathological hyperexcitability. Prominent cell loss and gliosis in CA1 is also occasionally seen in patients with AD and other dementias.^3,27^

**Treatment.** Epilepsy treatment in AD may pose a challenge because this type of patient is particularly vulnerable to the adverse effects of drugs with central nervous system activity as well as to drug interactions.^1,28,29^ Therefore, the objective of antiepileptic drugs (AEDs) treatment in this group takes into account several factors beyond adequate seizure control.^3,12,28,29^

Evidence demonstrates that the first-generation drugs, such as phenobarbital and phenytoin, should be avoided because, in addition to having a known effect on cognition, they can produce sleepiness and osteomalacia and pose a subsequent greater risk of fracture due to loss of balance and ataxia.^1,2^ In the case of valproic acid (VPA), it has not been useful in agitation in patients with AD and is also associated with cognitive decline, and likewise for benzodiazepines (BZDs). Carbamazepine (CBZ) may be difficult to use because of its cardiac effects, hyponatremia and sedation.^2,4^

Among the newer drugs, lamotrigine (LTG) presents a positive neuropsychological profile, including mood stabilization.^2^ Scant data is available regarding topiramate, however the associated reduction in attention and verbal fluency well-described in adults, suggests that this drug should be used with caution.^1,2^ Gabapentin (GBP) has no significant protein binding and no reported interactions, and its most common adverse effects include drowsiness, fatigue, and weight gain. There is conflicting evidence regarding an improvement in behavioral disorders in advanced AD with the use of GBP.^1^ Levetiracetam (LEV) has proven to be a good option because it presents linear pharmacokinetics, renal excretion (therefore less drug interaction), is broad spectrum (used in generalized and focal seizures), while some studies have also shown some degree of benefit in cognitive function.^28-30^ However it is important to mention that the main concerns with LEV are behavioral side effects, most commonly depression, with a prevalence of approximately 7%.^1^

Rowan et al. (1995) apud Hommet et al. (2008) conducted a study entailing a multicenter clinical trial of seizures in patients aged 65 and older, with newly diagnosed seizures. In the study, some of the patients had mild cognitive impairment (35%) or memory loss (26%), and LTG and GBP were compared to CBZ showing comparable efficacy, although patient tolerance was best with LTG, immediately followed by GBP.^2^

A prospective observational study following 25 patients with advanced AD and recent onset of epilepsy (onset in the last 3 months) in use of varying doses of LEV (1000 to 2000 mg/day) for a period of 14-25 months, showed that 18 out of the 25 patients (72%) responded to the treatment (established as a year without seizures), 4 (16%) discontinued use of the drug because of side effects (described as drowsiness, gait problems, psychomotor agitation and confusion).^28^

In order to evaluate the efficacy, tolerability, and cognitive effects of LEV in patients with AD and epilepsy, a prospective randomized case-control study with 95 patients taking LEV (n=38), phenobarbital (PB) (n=28), and LTG (n=29) was performed. After 4 months of dose adjustments, the patients were followed for 12 months. The three groups were compared with a control group (n=68). No difference in the effectiveness of the three drugs was evident, but LEV caused fewer adverse effects than other AEDs and was associated with improved cognitive performance, especially attention and verbal fluency.^29^

## DISCUSSION

AD patients have a higher risk of seizures and epilepsy than the general population. However, little is known about the influence of epilepsy on the evolution of AD or in terms of reducing cognitive abilities, autonomy and also increasing the risk of injury.

Determining the incidence and prevalence of the two disorders in combination runs into methodological problems, such as the inclusion of other types of non-AD dementia and the difficulty of establishing a reliable diagnosis of epilepsy because methods adopted vary widely among studies. There are few studies with pathological confirmation of AD. We can speculate that some of these patients had hippocampal sclerosis, predisposing to epilepsy, as opposed to AD, albeit with the same clinical picture.

Types of seizures are rarely reported in the literature, although partial seizures seem to be more frequent in this population. In a study involving a large number of patients with different etiologies of cognitive disorders, a small number of patients exhibited EEG abnormalities. EEG in patients with dementia and epilepsy may well be less important in the determination of type of epilepsy.

AD patients present with an accumulation of amyloid which could predispose them to neuronal excitability, and Hyperphosphorylated tau. In mice models these two anomalous proteins appear to play a role in the generation of seizures. In addition, neuronal loss and gliosis is evident in the hippocampus, factors which may contribute to the genesis of epileptic activity.

It is known that AEDs are associated with significant side effects and interactions with a large number of medications because of their ability to stimulate or inhibit hepatic metabolism of the cytochrome P450 enzyme. ^12,28^ Consequently, it is necessary to pay greater attention when choosing the drug because the elderly generally make use of multiple therapies for their comorbidities.^2,12,28,29^

Studies examining the use of AEDs, specifically in patients with AD, are scarce, hindering the development of guidelines for choosing the best medication in this group. Available data suggest that the new generation of drugs is better tolerated in the elderly population, and that LEV/LMT/GBP may prove to be a valuable therapeutic option. Studies comparing the efficacy and tolerability of AEDs in patients with epilepsy and AD are limited. Some data suggest that seizures due to AD are usually responsive to treatment.^16,28^

In the absence of specific studies with significant results, the choice of AEDs is still made empirically, considering the side-effect profile of each drug and the presence of concomitant symptoms (such as behavioral or depression) that could be better treated with an associated antiepileptic.

Finally, the body of information currently available on the subject needs to be furthered by means of well-designed studies with a greater number of participants, EEG monitoring and neuroimaging studies, so as to enable the adoption of better practices regarding treatment and prognosis. Further prospective studies with outcomes of concomitant dementia and epilepsy, neurophysiological findings and biomarkers should be performed.
